# CT/MRI LI-RADS v2018 vs. CEUS LI-RADS v2017—Can Things Be Put Together?

**DOI:** 10.3390/biology10050412

**Published:** 2021-05-06

**Authors:** Cosmin Caraiani, Bianca Boca, Vlad Bura, Zeno Sparchez, Yi Dong, Christoph Dietrich

**Affiliations:** 1 Department of Medical Imaging, “Iuliu Hațieganu” University of Medicine and Pharmacy Cluj-Napoca, 400012 Cluj-Napoca, Romania; cosmin.caraiani@umfcluj.ro; 2 Department of Radiology, County Clinical Emergency Hospital Cluj-Napoca, 400006 Cluj-Napoca, Romania; vb423@cam.ac.uk; 3 Department of Radiology, “George Emil Palade” University of Medicine, Pharmacy, Science and Technology of Târgu Mureș, 540139 Târgu Mureș, Romania; 4 Department of Radiology, Addenbrooke’s Hospital and University of Cambridge, Cambridge CB2 0QQ, UK; 5 Department of Gastroenterology and Hepatology, Regional Institute of Gastroenterology and Hepatology “Prof. Dr. Octavian Fodor”, 400158 Cluj-Napoca, Romania; 6 3rd Medical Department, “Iuliu Hatieganu” University of Medicine and Pharmacy, 400162 Cluj-Napoca, Romania; 7 Ultrasound Department, Zhongshan Hospital, Fudan University, Shanghai 200032, China; dong.yi@zs-hospital.sh.cn; 8 Department Allgemeine Innere Medizin (DAIM), Kliniken Hirslanden Beau Site, Salem und Permancence, 3013 Bern, Switzerland; christophfrank.dietrich@hirslanden.ch

**Keywords:** LI-RADS, ultrasound, CEUS, CT, MRI

## Abstract

**Simple Summary:**

The LI-RADS system is nowadays the mainstream system used in classifying liver nodules in cirrhotic liver according to their risk of malignancy. Two main LI-RADS documents have been released—the CEUS LI-RADS v2017 document, and the CT/MRI LI-RADS v2018 document. In some circumstances, a nodule can be differently classified when using CEUS versus when using CT or MRI. In this paper, we also focus on the existing similitudes between the two documents but, essentially, on the differences between the two main documents and the complementarities between imaging techniques in characterizing liver nodules in cirrhotic livers. Awareness of the complementarity of imaging techniques may lead to an improvement in the characterization and classification of liver nodules and will reduce the number of liver biopsies. This paper proposes practical solutions in order to better classify and manage observations or nodules detected in cirrhotic livers.

**Abstract:**

Different LI-RADS core documents were released for CEUS and for CT/MRI. Both documents rely on major and ancillary diagnostic criteria. The present paper offers an exhaustive comparison of the two documents focusing on the similarities, but especially on the differences, complementarity, and added value of imaging techniques in classifying liver nodules in cirrhotic livers. The major diagnostic criteria are defined, and the sensitivity and specificity of each major diagnostic criteria are presented according to the literature. The existing differences between techniques in assessing the major diagnostic features can be then exploited in order to ensure a better classification and a better clinical management of liver nodules in cirrhotic livers. Ancillary features depend on the imaging technique used, and their presence can upgrade or downgrade the LI-RADS score of an observation, but only as far as LI-RADS 4. MRI is the imaging technique that provides the greatest number of ancillary features, whereas CEUS has fewer ancillary features than other imaging techniques. In the final part of the manuscript, some recommendations are made by the authors in order to guidephysicians as to when adding another imaging technique can be helpful in managing liver nodules in cirrhotic livers.

## 1. Introduction

**Figure 1:**
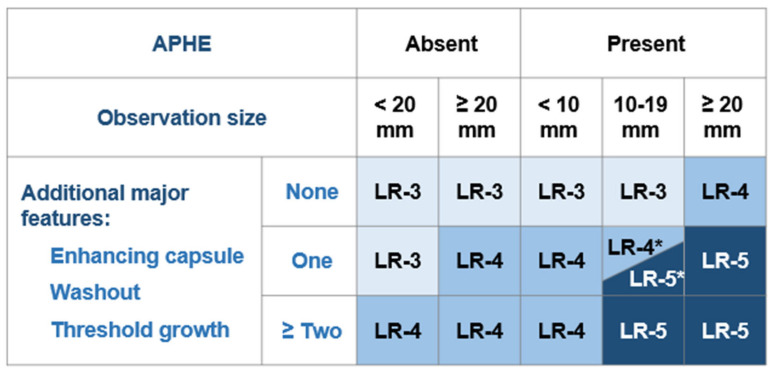
CT/MRI LI-RADS diagnostic table, adapted from CT/MRI LI-RADS^®^ v2018 core document. * LR-4 if the one additional feature is an enhancing capsule, otherwise, LR-5; APHE—arterial phase hyperenhancement (as defined within the text, non-peripheral).

**Figure 2:**
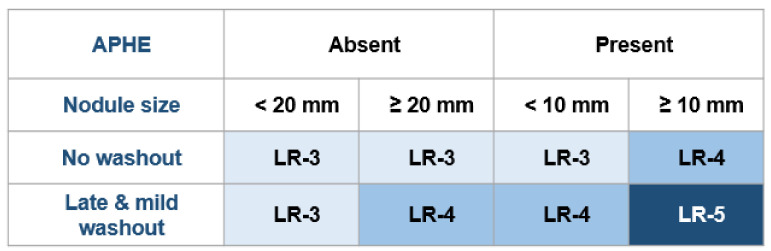
CEUS LI-RADS diagnostic table, adapted from CEUS LI-RADS^®^ v2017 core document. APHE—arterial phase hyperenhancement (as defined within the text, not rim, nor peripheral discontinuous globular).

According to current guidelines, hepatocellular carcinoma (HCC) can be diagnosed in patients with cirrhosis based solely on radiologic hallmarks, without the need for histologic confirmation [1,2]. The liver imaging reporting and data system (LI-RADS) was created to standardize liver imaging and reporting in patients at risk for HCC. It assigns probabilities for a nodular hepatic lesion to be HCC, benign, or non-HCC malignancy. The LI-RADS criteria are in use for contrast-enhanced computed-tomography (CT), contrast enhanced magnetic resonance imaging (MRI) (Figure 1), and contrast enhanced ultrasound (CEUS) (Figure 2). Different core documents were issued for CT/MRI and, CEUS, respectively [3,4]. The last CEUS-LIRADS version was released in 2017, whereas the last LI-RADS version for CT/MRI was released in 2018. Both documents describe major and ancillary imaging features (AF) of HCC in cirrhotic patients. CEUS-LI-RADS is only considered appropriate for HCC diagnosis, whereas CT/MRI LI-RADS can be used for both diagnosis and staging of HCC [3,4].

For CT and MRI, the 2018 version of LI-RADS brings the following as new elements (as compared to the 2017 version):

- Threshold growth, a major diagnostic criterion, has a simplified definition in the latest document. Now, it only refers to an increase in size of over 50% of an observation in less than 6 months. A new observation of ≥10mm, or a ≥100% increase in size of an observation over more than 6 months are now considered criteria for subthreshold growth, which represents an ancillary feature.
- In order to simplify the LI-RADS algorithm, LI-RADS 5g and LI-RADS 5us categories were eliminated. In practice, this refers to observations with arterial phase hyperenhancement (APHE), with a size ≥10 mm and ≤19 mm. In the previous 2017 version, ultrasound visualization of a nodule was necessary for observations measuring 10 to 19 mm with APHE and non-rim washout in order to categorize the observation as LI-RADS 5 (LI-RADS 5us). If the observation was not visible by ultrasound, the nodule was classified as LI-RADS 4. In the new document, every observation measuring 10 to 19 mm with APHE and non-rim washout can be classified as LI-RADS 5. Observations with a size ≥10 mm and ≤19 mm with APHE and threshold growth (defined as the mentioned above) are now classified as LI-RADS 5, not LI-RADS 5g, as previously. Observations measuring 10 to 19 mm with APHE and an enhancing capsule, and with no non-rim washout and/or threshold growth, are classified in both documents as LI-RADS 4 [5].

This paper compares the two main LI-RADS core documents, highlighting similarities and differences between them. Situations when imaging techniques are complementary are also highlighted and improvements in the classification of liver nodules after the combined use of imaging techniques are suggested. All the major and some of the ancillary HCC features will be discussed and presented from both documents’ perspective.

In the literature, there are other papers comparing LI-RADS CEUS 2017 with the previous LI-RADS CT/MRI document. [6,7]. However, they more on the differences between the two main documents and not on their complementarity nor on the added value of imaging techniques, which are the focus of the current paper.

Some scientific papers focus on the contribution of CEUS to accurately classify indeterminate observations on CT/MRI or hypovascular nodules with a LI-RADS 3 or LI-RADS 4 appearance on sectional imaging [8,9]. However, as far as we know, our current paper is the first one exhaustively comparing the two LI-RADS core documents by comparing the reported accuracy of techniques in assessing each major and each ancillary feature that is common in both documents. Moreover, we propose practical solutions derived from the complementarity of the imaging techniques (CEUS versus CT/MRI) in order to better classify and manage observations or nodules detected in cirrhotic livers.

## 2. Observation or Focal Liver Lesion

### 2.1. Definition

Firstly, we need to introduce the LI-RADS term “observation”, which is a distinctive area compared to the background liver at imaging [10]. An observation may represent a true lesion (if there is a corresponding pathologic abnormality) or a pseudolesion (if there is not). The term “observation” only applies to CT and MRI, but not to CEUS. As a generic LI-RADS term, it is preferred over focal liver lesion (FLL) or nodule, since some observations (e.g., perfusion alterations, artifacts) may represent pseudolesions rather than true lesions or nodules. In the published literature on ultrasound and CEUS, the term “observation” is rarely used. The World Federation for Ultrasound in Medicine and Biology (WFUMB) and European Federation of Societies for Ultrasound in Medicine and Biology (EFSUMB) guidelines on CEUS of the liver prefer the term “FLL” over “nodule”.

### 2.2. Phases of Enhacement

The main criterion for characterizing an FLL is its behavior following intravenous contrast injection. For a proper characterization of FLLs, a tri-phasic evaluation is mandatory: arterial, portal, and late phases are defined by the time gap between contrast injection and image acquisition. While CEUS offers a continuous, real-time evaluation of a lesion’s behavior, CT and MRI only immortalize static frames of multiple time-points following contrast injection.

Regardless of the technique used, the arterial phase represents the acquisition immediately following the moment of contrast injection. For CT/MRI diagnosis and characterization of focal liver lesions, particularly HCC, a late arterial phase is preferred. Images are thus acquired 35 to 45 s after contrast media injection [3]. In CEUS, the arterial phase starts at 10–20 s and ends 30–45 s after contrast injection [4].

On CT/MRI, the portal phase is characterized by image acquisition at 60–75 s after contrast injection. The portal venous system is completely visualized and the liver parenchyma is at its peak enhancement [3]. In CEUS, the portal phase starts at 30–45 s and lasts for up to 120 s following contrast injection [4].

The late (delayed) phase image acquisition on CT/MRI occurs at any point between 2 to 5 min following contrast administration. Contrast is still visible within the portal venous system and liver parenchyma, but is not as prominent as during the portal phase. With CEUS, the late phase lasts from 2 to 6 min after contrast injection [3,4].

Some contrast media used in MRI (called hepatobiliary agents, as their excretion is not only through kidney but through liver as well) can offer us a very late hepatobiliary phase. In this phase, the liver parenchyma enhances more than the blood vessels and the contrast media is also excreted into the biliary tree.

## 3. Major Features

An overview of the major LI-RADS features compared between CEUS and CT/MRI is presented in Table 1.

### 3.1. Arterial Phase Hyperenhacement (APHE)

#### 3.1.1. Definition

Arterial phase hyperenhancement (APHE) is the most important feature in characterizing HCC in patients at risk for liver cirrhosis. Therefore, an observation cannot be defined as LI-RADS 5 without APHE, regardless of size or any other features [11]. In LI-RADS, APHE is defined as non-rim like enhancement of the entire or part of an observation, unequivocally greater than the enhancement of the surrounding liver [12]. Rim enhancement and rim washout, only involving the periphery of a lesion, suggests a non-HCC malignancy (and is therefore defined as LI-RADS M). APHE has good sensitivity for the diagnosis of progressed HCC (reported by different papers as ranging between 65 and 96%), but may be absent in well-differentiated HCC [12]. Studies reporting the presence of APHE on CT and MRI scans of pathology-proven HCC show a rather mediocre sensitivity of APHE for HCC diagnosis (74%)—which is even lower (43 to 53%) for lesions smaller than 1 cm [13].

#### 3.1.2. Comparison of CEUS, CECT, and MRI, Similarities and Differences

##### Similarities

In both documents, APHE is defined as greater enhancement of an observation as compared to the surrounding liver. APHE can be diffuse, throughout the whole observation, or limited to part of the observation. Both documents exclude rim enhancement, which is defined as arterial phase hyperenhancement more pronounced in the periphery of the lesion and suggests malignancy in general, other than HCC [12]. Peripheral discontinuous globular APHE is also excluded, as it is specific for hemangiomas.

##### Differences and Complementarity of Techniques

**Figure 3:**
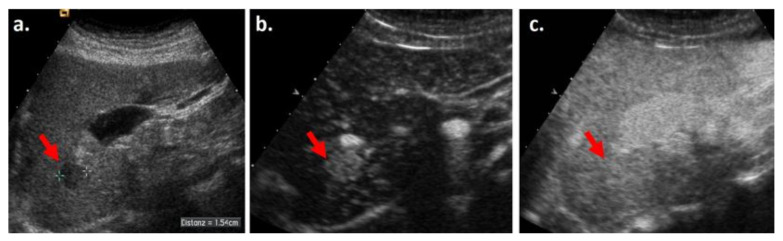
Grayscale ultrasound of the liver showing a slightly hypoechoic nodule in segment six of the liver in a cirrhotic patient (**a**). On CEUS, the nodule was hypervascular in the arterial phase (**b**) with no washout in the late phase (**c**), and it was consequently classified as LI-RADS 4. On CT, the nodule was isoenhancing to the liver parenchyma in all phases of enhancement. The nodule was a biopsy proven HCC.

The use of CEUS can improve the sensitivity of APHE for HCC diagnosis. CEUS provides real-time enhancement of a lesion, whereas on CT or MRI, the lesion is visualized in most cases during the late arterial phase only. There are several studies that have analyzed the value of adding CEUS for indeterminate, arterial phase non-hyperenhancing (APNHE) liver lesions at MRI or CT [14]. Their results showed that 25.9% and 27.9%, respectively, of APNHE observations further presented APHE in CEUS examination, all of them being diagnosed as HCC [9,14]. Moreover, Takayasu et al. reported a higher sensitivity of APHE for well-defined HCC on CEUS than on MRI (59.3% vs. 46.3%), while the results of Bolondi et al. showed that CEUS provides more sensitive detection of APHE than CT for HCC observations (APHE sensitivity on CEUS = 88% vs. 77% on CT) [15,16]. CEUS can therefore depict APHE in lesions with no definite APHE on CT and MRI (Figure 3). Such lesions that would, at most, be characterized as LI-RADS 4 (no APHE depiction) on CT/MRI would be upgraded from LI-RADS 3 to 4 or from LI-RADS 4 to 5 by CEUS [6]. The only exception to this is a lesion less than 20 mm in size, with no other HCC feature—which would be characterized as LI-RADS 3, irrespective of APHE presence.

The presence of hypervascular pseudolesions such as arterio-portal shunts will manifest on CT and MRI as APHE, lowering the specificity of APHE for HCC [17]. CEUS generally targets lesions identified on baseline ultrasound scans. Since hypervascular pseudolesions are not seen on a baseline scan, they would not be depicted on CEUS. Therefore, the positive predictive value of CT/MRI APHE for HCC is rather mediocre (ranged between 65 and 81%) [18,19,20], whereas the positive predictive value of CEUS is significantly higher. Several studies reported a positive predictive value (PPV) value of 100% of CEUS APHE for the diagnosis of HCC [21,22]. Additionally, the specificity of APHE detected on CEUS is very high for the diagnosis of HCC, reaching 100% [21,22]. PPV and specificity are closely related. Consequently, any observation with APHE on CEUS measuring 10 mm or more showing APHE is either an LI-RADS 4 or an LI-RADS 5 nodule [6]. In contrast, a lesion depicted on CT/MRI, measuring 10 to 20 mm with APHE only and no other major HCC features, is considered, according to CT/MRI LI-RADS core document, only a LI-RADS 3.

### 3.2. Washout

#### 3.2.1. Definition

Washout is defined as a temporal reduction in the enhancement of an observation in whole or in part, relative to the surrounding liver parenchyma, from an earlier to a later phase, resulting in hypoenhacement [3,4]. LI-RADS prefers washout over hypoenhancing because the degree of enhancement of the observation during the late phases has to be unequivocally lower than that in the earlier phases (not only unequivocally lower than the enhancement of the liver parenchyma) in order to be a major LI-RADS criteria [23].

#### 3.2.2. Comparison of CEUS, CECT and MRI, Similarities and Differences

##### Similarities

- Washout represents a major imaging feature in both CT/MRI LI-RADS and CEUS LI-RADS cores, its presence excluding a LR-1 and LR-2 observation
- However, the sole presence of washout is not sufficient for an observation to be categorized as LR-5 on either CEUS or CT/MRI. There are a few papers that investigated the performance of washout as a standalone feature for the diagnosis of HCC and the reported specificities ranged between 62–100%. Furthermore, in all of these studies, the combination of “washout” and APHE has proved to have higher specificity (96–100%) and PPV (97–100%) when compared with the specificity of “washout” alone [24,25,26].
- On CEUS, as well as on CT/MRI, washout can be applied for any enhancing observation, even in the absence of APHE.

##### Differences and Complementarity of Techniques

**Figure 4:**
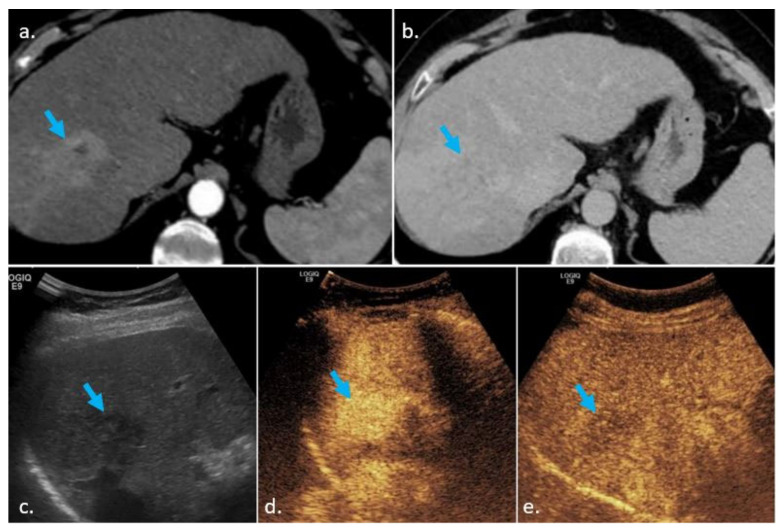
CT scans of the liver obtained in the arterial (**a**) and late phases (**b**). The blue arrow shows a hypervascular nodule (**a**) with washout (**b**) localized in segment two of the liver, close to the left portal vein. On CT, the nodule was classified as LI-RADS 5. On US (**c**), the nodule (blue arrow) was slightly hyperechoic/isoechoic as compared to the surrounding liver parenchyma. On CEUS, the blue arrow shows the same nodule, which presented APHE (**d**) but did not show any washout (**e**), therefore, it was classified as LI-RADS 4. This is a rather atypical behavior, as washout is more often seen on CEUS as compared to CT/MRI.

**Figure 5:**
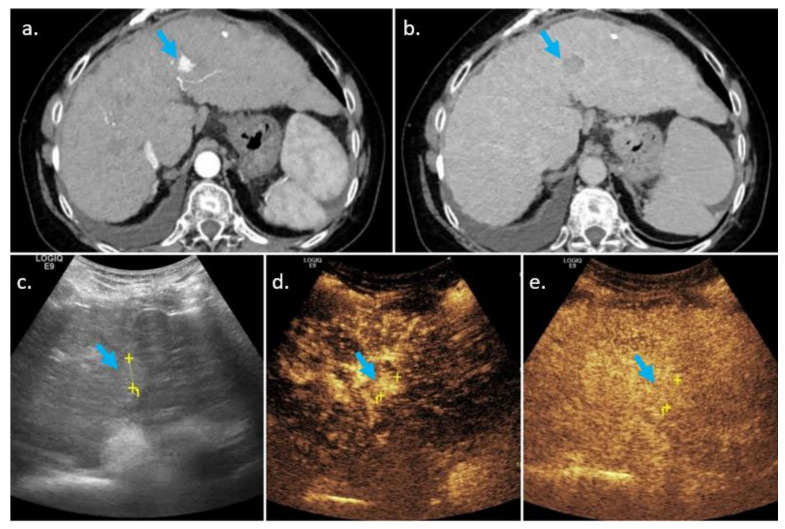
CT scans of the liver obtained in the arterial (**a**) and late phases (**b**). A hypervascular nodule (blue arrow) is seen in segment eight of the liver (**a**). In the late phase (**b**), the nodule becomes isoenhancing to the liver parenchyma (blue arrow). The nodule was consequently classified as LI-RADS 4 on CT. On US (**c**), the nodule was slightly hypoechoic/isoechoic (blue arrow) as compared to the surrounding liver parenchyma. On CEUS, the nodule (blue arrow) presented APHE (**d**) and late and mild washout (**e**), and was classified as LI-RADS 5.

Several papers showed that the presence of washout is more often encountered in HCC on CEUS, as compared to CT/MRI. A recent study by Wang et al. reported a washout appearance observed more frequently on CEUS than on CE-MRI (50% vs. 28.6%) [27]. Additionally, the results of Hu et al. showed that 38% of HCC nodules without washout on MRI presented further washout when evaluated on CEUS [8]. Nodules which can be characterized as LI-RADS 5 on CEUS (APHE and the presence of late and mild washout) would be LI-RADS 4 or even 3 at CT/MRI (due to the absence of washout). One possible explanation for this is the difference in contrast agents used in CEUS versus CT/MRI (Figure 4 and Figure 5).

Ultrasound contrast is a pure “blood pool” agent, which remains intravascular, not passing the vascular endothelium [28]. On the other hand, CT/MRI contrast agents can extravasate into the tumor interstitium, resulting in the gradual enhancement of malignant lesions during the late phase [29,30,31]. There are several differences between the CT/MRI LI-RADS core and the CEUS LI-RADS core concerning the presence or absence of washout:

- Washout versus “washout”

CEUS LI-RADS core uses the term washout for characterizing an observation that reduces its enhancement, while on CT/MRI LI-RADS core, the correct term is “washout”. This difference happens since, on CEUS, the washout phenomenon is a true one, while on CT/MRI, the washout of an observation may only be apparent, and can actually be the result of an increased enhancement of surrounding liver tissue, rather than an actual reduction in a nodule enhancement.

- The characterization of washout by its onset and degree

CEUS LI-RADS document divides washout into (a) late (>60 s) and mild washout (its presence is a major criterion for HCC) and (b) early (<60 s) and/or strong washout (which categorizes a liver observation as LI-RADS M or non-HCC malignancy). Conversely, washout is a major HCC criterion on CT/MRI regardless of its intensity or onset. In the last few years, there have been several published papers that investigated the importance of washout onset and degree for the differentiation between HCC and other malignancies in CEUS [12,32,33,34,35,36]. All these studies reported that the majority of intrahepatic cholangiocarcinoma (ICC) or other non-HCC malignancies showed an early and marked washout, usually within 1 min after contrast injection, while the majority of HCC were characterized by a late and mild washout. Moreover, Terzi et al. demonstrated that if the washout criterion was applied based only on its presence and regardless of its onset and degree, the PPV for HCC would have been lower: 94% versus 98.5% [37].

Therefore, in CEUS LI-RADS core, an observation should be categorized as LI-RADS M (LR-M) if it has one of the following features: rim APHE or early or marked washout. However, it should be noted that all LR-M lesions should be histologically proven. Still, a significant percentage (48%) of LR-M lesions on CEUS are atypical HCC at biopsy, rather than non-HCC malignancies [6].

- The characterization of washout by its spatial pattern

In CT/MRI LI-RADS document, “washout” as a major imaging feature specific for HCC, which requires the presence of a non-peripheral temporal reduction in enhancement in a liver observation, while a peripheral “washout” is a characteristic feature for LR-M lesions. In CEUS LI-RADS, the washout criterion does not mention its localization within the liver observation.

This difference occurs because of the type of contrast agents used for the imaging modalities and their different tissue behavior and kinetics. The purely intravascular contrast microbubbles used for CEUS are drained rapidly from all tumor compartments, including the fibrotic center of ICC, resulting in a hypoechoic appearance of the entire liver observation, regardless of its tissue components. Conversely, the contrast agents used for CT/MRI drain rapidly from the arterialized, peripheric part of a lesion, but they accumulate gradually into the centrally located interstitium. The ICC and other non-hepatocellular malignancies with a central fibrotic component will appear as targetoid observations with peripheral hypoenhancement and central delayed enhancement [38,39,40,41,42]. However, the peripheral “washout” is encountered especially in ICCs >3 cm, while the small ICCs often exhibit non-peripheral “washout” [43,44,45]. Moreover, some HCCs may present peripheral “washout”. Therefore, the differential diagnosis based only on this spatial criterion may be sometimes difficult.

### 3.3. Threshold Growth

#### 3.3.1. Definition

The definition for threshold growth was simplified in version 2018 of the CT/MRI core document, achieving concordance with American Association for the Study of Liver Diseases (AASLD) and Organ Procurement and Transplantation Network (OPTN) definitions. Threshold growth is clearly defined as an increase in size ≥50% in ≤6 months, and it only represents a major feature favoring malignancy in the CT/MRI LI-RADS core document, but not in the CEUS core document.

#### 3.3.2. Comparison of CEUS, CECT, and MRI, Similarities and Differences

##### Similarities

Growth is viewed and defined differently within the two core documents. Regardless of the imaging techniques used, the radiologist should use the same plane as prior exam to assess growth, on the same imaging mode in CEUS (or B-mode ultrasound) and the same phase and sequence on serial imaging (CT/MR).

##### Differences and Complementarity of Techniques

Regarding the threshold growth, there are the following differences between CT/MRI LI-RADS and CEUS LI-RADS core documents:

- Threshold growth is a major feature for HCC in the CT/MRI LI-RADS core document, but only an ancillary feature suggesting malignancy in the CEUS core document.
- Definite growth is defined by the CEUS LI-RADS core document as the unequivocal increase in size of a lesion; there is no established “threshold”, but >5 mm is generally considered unequivocal growth. Ultrasound should only be compared with ultrasound and the size increase should not be attributable to artifacts, measurement errors, or difference in technique [46].
- Unequivocal growth evaluated by CEUS favors malignancy in general, not HCC in particular (as threshold growth does in CT/MRI).
- Using the arterial phase of enhancement when measuring an observation should be avoided, if possible, on CT/MRI due to the risk of overestimating the lesion size. On CEUS, measuring the observation can, in most cases, only been done in the arterial phase. On CT/MRI, an observation should be measured in the phase, sequence, and plane in which its margins are the most clear. In the meantime, measuring a lesion in the arterial phase or on diffusion weighted imaging should be avoided [47].
- Threshold growth is considered of less importance in CEUS as compared to CT/MRI. This is because of the lesser reproducibility of US images as compared to CT/MRI and the difficulties of obtaining the same plane of the lesion on seriate US examinations [6].

Increase in size of a lesion is a criterion with good specificity for the diagnosis of HCC, threshold growth (evaluated by CT/MRI) having high specificity—between 83–85% [48]. This means that if one nodule increases in size more than 50% over less than 6 months, it is much more likely to be HCC. Various studies reported HCC tumor volume doubling time ranging from as low as 9 days [11] to more than a year [49,50,51]. The growth rate depends on the degree of differentiation; well-differentiated HCC tends to grow slower, while moderate and poor differentiated HCC have shorter doubling times [52,53,54]. However, any increase in size of a nodule is considered an ancillary feature on CEUS examination [4]. Therefore, we recommend associating CT or MRI in every patient in which US/CEUS suggests increase in size of a nodule—unequivocal threshold growth associated with APHE can classify the nodule as LI-RADS 5 and biopsy can be avoided.

### 3.4. Enhancing Capsule

An enhancing capsule is a major diagnostic feature in the CT/MRI core but not in the CEUS core. Capsule presence is defined as a uniform, sharp border, thicker than the fibrotic tissue of the background nodules, which is detected as an enhancing rim in the portal, late, or transitional phase [12]. A capsule should be differentiated from “rim enhancement”, which is characteristic for LI-RADS M: rim enhancement is typically pronounced in the arterial phase with later washout, whereas a capsule is enhanced in the portal or late phase and is enhanced less or equally than the surrounding liver in the early phases [55]. The differences between CEUS and CT/MRI in depicting the capsule may be due to the fibrotic content of the capsule as CT/MRI contrast media will diffuse into interstitial tissue, in contrast to ultrasound contrast media, which remains strictly intravascular.

According to previous MRI studies, the presence of an enhancing capsule has low/moderate sensitivity, ranging between 32.9–55%, and very high specificity, within the range of 83–98.8% for the diagnosis of HCC [26,56,57]. Regarding the differences between CT and MRI, Zhang et al. showed that, when compared to MRI, CT produced false-negative findings of a pseudocapsule by 42.9%, thus underestimating the LI-RADS score of liver lesions [58]. Additionally, the study of Corwin et al. revealed that nearly half of the liver observations had the LI-RADS category upgraded upon MRI compared with CT, one of the reasons for this being the visualization of a delayed enhancing capsule on MRI, which was not seen on CT [59]. Another study, which evaluated the value of LI-RADS features on contrast-enhanced CT for the diagnosis of HCC, showed that an enhancing capsule had the lowest sensitivity (20.7%) among the major features [48].

## 4. Ancillary Features

### 4.1. Definition

Ancillary features (AF) are imaging features, which modify an observation’s likelihood of being HCC. AF favoring HCC can upgrade the LI-RADS score of an observation by one category, up to LI-RADS 4. AF cannot upgrade an observation from LI-RADS 4 to LI-RADS 5; therefore, for LI-RADS 5, a combination of major features is needed. AF favoring benignity will downgrade the LI-RADS score by one category. The absence of AF favoring malignancy or benignity should not be used neither to downgrade nor upgrade the LI-RADS score, respectively.

### 4.2. Comparison of CEUS, CECT and MRI, Similarities and Differences

The similarities and differences between AF that can be evaluated on both CEUS and CT/MRI are summarized in Table 2.

#### 4.2.1. Similarities

Although AF differ depending on the modality used, the way they are applied to modify the LI-RADS score is similar for CEUS, CT, or MRI. AF are to be used at the radiologist’s discretion for improved detection, increased confidence, or category adjustment. An ancillary feature should be characterized as absent if its presence is uncertain. AF favoring benignity will downgrade the LI-RADS score by one category, while AF favoring malignancy will upgrade the score by one category. The LI-RADS score cannot be upgraded to 5 with AF in either technique [3,4].

Ancillary features are classified, in both documents, in the following:

- AF favoring malignancy in general
- AF favoring HCC in particular
- AF favoring benignity

The ancillary features which are common for the two documents are as follows:

- The mosaic architecture and nodule in nodule (both considered AF which favor HCC in particular)
- Stability in the size of an observation ≥2 years in the absence of treatment or unequivocal decrease in size of a lesion (both considered as AF favoring benignity)

Mosaic appearance and nodule in nodule architecture are AF in both core documents. Nodule in nodule architecture is considered to be a subtype of mosaic appearance [45]. Mosaic architecture refers to the presence of randomly distributed internal nodules and compartments inside a liver nodule, usually with different imaging features. Heterogeneity of a liver mass is more easily depicted on MRI as compared with CT/CEUS, and on MR T2 weighted sequences as compared to T1 weighted sequences. A mosaic pattern is seen in 28–63% of HCC nodules with a size greater than 3 cm [60].

The nodule in nodule architecture corresponds to the presence of a smaller, inner nodule with different imaging features than the larger outer nodule [61]. In many cases, a nodule in nodule appearance can correspond to a smaller HCC nodule developing into a bigger dysplastic nodule [62].

**Figure 6:**
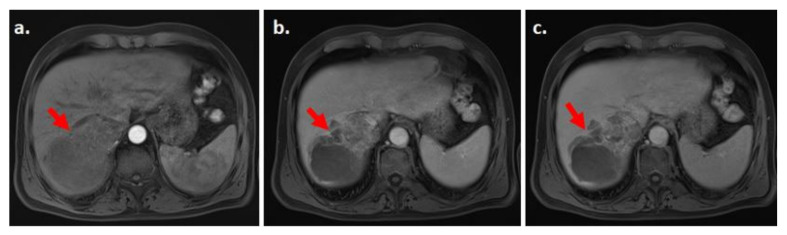
MRI of the liver. Nodule in segment seven (red arrow), which shows progressive enhancement in the arterial (**a**), portal (**b**), and late phase (**c**). The nodule was classified as LI-RADS M on MRI and the radiologist suspected a cholangiocarcinoma. On CEUS, the lesion had a “nodule in nodule” architecture and the inner nodule showed APHE and washout, and the nodule was classified as LI-RADS 5.

If the inner nodule (in the case of nodule in nodule “architecture”) or one part of the inner structure of the lesion displays LI-RADS 5 features (such as APHE and washout), the whole observation should be considered LI-RADS 5 and the aspect should not be interpreted as an ancillary feature [63] (Figure 6).

#### 4.2.2. Differences

AF differ depending on the modality used.

Regarding LI-RADS score assessment, CEUS provides fewer AF than other modalities:

- Interval growth of an observation

Unequivocal size increase is an AF that favors malignancy in general, whereas size stability (for at least 2 years and in the absence of treatment) or unequivocal size reduction of a lesion are AF which favor benignity.

- Nodule in nodule architecture (favors HCC)
- Mosaic architecture (which equally favors HCC)

The LI-RADS CT/MRI v2018 core document provides additional AF, not assessable by CEUS. Some of those features can be evaluated by both CT and MRI, while others are applicable only to MRI. These are listed in Table 3; defining them goes beyond the scope of this article, and we kindly refer the reader to the CT/MRI LI-RADS v2018 core document for that purpose [3].

In the CEUS LI-RADS document, any increase in size of an observation is an AF favoring malignancy. In the CT/MRI LI-RADS document, an increase in size of an observation is divided into the following:

- threshold growth (increase of a mass by ≥50% in ≤6 months)
- subthreshold growth is defined as increase in size of an observation by less than 50% in 6 months, by any size increase in more than 6 months, or by the appearance of a new lesion, regardless of its size [41].

A new observation in a cirrhotic liver cannot be considered HCC (LI-RADS 5) without other diagnostic criteria, which may include the lesion as LI-RADS 5. On MRI examination, subthreshold growth as a standalone feature has a sensitivity of 48% and a specificity of 91% for the diagnosis of HCC [56]. A study of Alhasan et al., which evaluated the diagnostic performance of LI-RADS features onCT, reported a sensibility of 50.8% and a specificity of 66.9% of subthreshold growth for HCC [48].

##### Ultrasound Visibility as a Discrete Nodule

US visibility as a discrete nodule is an AF mentioned in the CT/MRI LI-RADS core and refers to visibility at non-enhanced US of an observation depicted by CT and MRI. As most of the benign lesions on a cirrhotic liver and all vascular pseudolesions are not to be seen, indistinguishable from the surrounding liver on B-mode ultrasound, most nodules seen by the means of ultrasound in a cirrhotic liver, are malignant [64]. A paper by Darnell et al. shows that 96% of LI-RADS 4 and 69% of LI-RADS 3 observations were HCC nodules if visible on ultrasound [65]. This data suggests that ultrasound visibility as a discrete nodule, is a strong AF favoring HCC.

## 5. Summary—Complementarity and Added Value of the Techniques

CEUS is considered appropriate for the diagnosis of HCC nodules, whereas CT/MRI are appropriate for both the diagnosis and staging of HCC. Even if a nodule is diagnosed as HCC on CEUS, a CT or MRI of the liver has to be performed before therapy in order to accurately stage the disease. This is due to the difficulties that US has in exploring the whole liver. For instance, obese or non-cooperating patients, a limited acoustic window, parenchymal heterogeneity, or reduced beam penetration represent possible limitations, which lead to inadequate evaluation of the entire liver [66]. Additionally, the location of the liver nodules is another concern, with two studies reporting that deep-seated, subdiaphragmatic lesions were difficult to visualize and assess on CEUS [67,68]. When dealing with multifocal disease, CEUS can only target one lesion in a short-lasting arterial phase, which represents another limitation of the technique. CT and MRI evaluate the whole liver parenchyma and multiple lesions can be characterized in a single-phase acquisition. Therefore, international guidelines do not recommend US or CEUS for staging HCC or for assessing the presence of metastasis [4].

- APHE is a crucial diagnostic feature of HCC. A liver nodule cannot be diagnosed by means of imaging as a LI-RADS 5 observation without APHE. APHE is more easily and accurately depicted by CEUS as compared to CT/MRI [9,14,15,29]. This means that, in practice, a nodule characterized as LI-RADS 3 or 4 by CT/MRI (e.g., a nodule without APHE presenting some washout) can be characterized as LI-RADS 5 by CEUS. We recommend CEUS in suspicious nodules without APHE on CT/MRI.
- CEUS is more sensitive than CT/MRI for depicting washout. In nodules with APHE but without washout on CT and MRI (LI-RADS 3 or 4), CEUS can prove the presence of washout, upgrading the nodule to LI-RADS 5 and, by this, avoiding biopsy. We recommend CEUS in nodules with APHE, but without washout on CT/MRI. On the contrary, if the observation, presenting only with APHE on CT/MRI, is not seen on US/CEUS, it is more likely a vascular pseudolesion and can be confidently considered as benign.
- Washout on CEUS was further divided into early and strong washout (characteristic of non-HCC malignancy) and late and mild washout (a major criterion for HCC). The rationale was improving the sensitivity of diagnosing non-HCC malignancies (particularly ICC) in cirrhotic livers. Still, by using these criteria, many atypical HCC nodules will have a LI-RADS M appearance on CEUS. CT and MRI can change the LI-RADS score for some of this nodules to LI-RADS 5 in these patients, avoiding biopsy, or increase confidence in the diagnosis of LI-RADS M by demonstrating other diagnostic features such as late phase central enhancement.
- Increase in size of a lesion is a criterion with good specificity for the diagnosis of HCC [48]. Threshold growth on CT/MRI is defined by an increase in size of one nodule of more than 50% over less than 6 months; however, any increase in size of a nodule is considered an ancillary feature on CEUS examination [4]. Therefore, we recommend associating CT or MRI in every patient for whom US/CEUS suggests increase in size of a nodule—unequivocal threshold growth associated with APHE can classify the nodule as LI-RADS 5 and biopsy can be avoided.
- For mosaic and nodule in nodule lesions, if APHE cannot be demonstrated by CT/MRI, we recommend additional imaging by CEUS, which is more sensitive in depicting APHE (and subsequently possibly classifying the lesion as LI-RADS 5).
- Ultrasound visibility of the observation as a discrete nodule is an ancillary feature, which helps in differentiating true hepatic lesions from vascular pseudolesions. In most cases, if used as an AF, it upgrades the LI-RADS score from 3 to 4. Many LI-RADS 3 nodules and a vast majority of LI-RADS 4 nodules depicted by ultrasound prove to be HCC. Therefore, if a LI-RADS 3 nodule is depicted by CT or MRI and was not described in the screening by surveillance ultrasound, we suggest repeating a targeted US, as sensitivity of screening ultrasound is known to be moderate, ranging between 58–94% for HCC detection at any stage [69,70,71,72,73], being even lower for the detection of early stage tumors, between 47–63% [71,72]. For LI-RADS 4 nodules, repeating US will not be necessary, as an ancillary feature cannot upgrade the score to LI-RADS 5.

## 6. Conclusions

CEUS, CT, and MRI are all established techniques in the diagnosis of focal liver lesions in cirrhotic livers. CEUS is appropriate only for HCC diagnosis, whereas CT and MRI are appropriate for both the diagnosis and staging of HCC. Nevertheless, CEUS still holds some advantages over CT/MRI, providing a more accurate evaluation of APHE and washout. Washout is divided in CEUS into strong and early washout and late and mild washout in order to differentiate HCC from non-HCC malignancies. On the other hand, the size increase of a lesion is more accurately evaluated on CT/MRI as compared to CEUS.

## Figures and Tables

**Table 1:** Major features within the CT/MRI LI-RADS^®^ v2018 core document—summary table for comparison across CEUS, CT, and MRI (similarities/differences).

	CEUS	CT/MRI
APHE	++ Real-time evaluation	+ Late phase arterial
Washout	++ True washout Late and mild (> 60 s)	+ Relative “washout” Regardless of intensity/onset
Threshold growth	Not a major feature CT/MRI recommended if positive	++
Enhancing capsule	Not appreciable	+/++

**Table 2:** Ancillary features (AF) presented in the CEUS LI-RADS^®^ v2017 and CT/MRI LI-RADS^®^ v2018 core documents—summary table for comparison across CEUS, CT, and MRI (similarities/differences). AF only assessable by CT/MRI were not included in this table.

		CEUS	CT	MRI
Favoring HCC	Mosaic appearance	+	+	++
	Nodule in nodule	+	+	++
Favoring malignancy (in general)	Size increase	Definite growth (+)	Subthreshold growth (+)	
Favoring benignity	Size stability >2 years	+	+	+
	Size reduction	+	+	+

**Table 3:** Ancillary features (AF) presented only in the CT/MRI LI-RADS^®^ v2018 core document.

Favored Diagnosis	Ancillary Feature	CT	MRI
Favoring malignancy (in general)	US visibility as discrete nodule	+	+
	Subthreshold growth	+	+
	Corona enhancement	+	+
	Fat sparing in solid mass	+/−	+
	Restricted diffusion	—	+
	Mild–moderate T2 hyperintensity	—	+
	Iron sparing in solid mass	—	+
	Transitional phase hypointensity	—	+
	Hepatobiliary phase hypointensity	—	+
Favoring HCC	Nonenhancing “capsule”	+/−	+
	Fat in mass, more than adjacent liver	+/−	+
	Blood products in mass	+/−	+
Favoring benignity	Parallels blood pool enhancement	+	+
	Undistorted vessels	+	+
	Iron in mass, more than liver	+/−	+
	Marked T2 hyperintensity	—	+
	Hepatobiliary phase isointensity	—	+

## Data Availability

Not applicable.

## References

[1] Bruix J., Sherman M. (2011). Management of hepatocellular carcinoma: An update. Hepatology.

[2] Bota S., Piscaglia F., Marinelli S., Pecorelli A., Terzi E., Bolondi L. (2012). Comparison of international guidelines for noninvasive diagnosis of hepatocellular carcinoma. Liver Cancer.

[3] American College of Radiology CT/MRI Liver Imaging Reporting and Data System Version 2018. https://www.acr.org/Clinical-Resources/Reporting-and-Data-Systems/LI-RADS/CT-MRI-LI-RADS-v2018.

[4] American College of Radiology CEUS LI-RADS^®^v2017 CORE. https://www.acr.org/-/media/ACR/Files/RADS/LI-RADS/CEUS-LI-RADS-2017-Core.pdf.

[5] Kielar A.Z., Elsayes K.M., Chernyak V., Tang A., Sirlin C.B. (2019). LI-RADS version 2018: What is new and what does this mean to my radiology reports?. Abdom. Radiol..

[6] Kim T.K., Noh S.Y., Wilson S.R., Kono Y., Piscaglia F., Jang H.J., Lyshchik A., Dietrich C.F., Willmann J.K., Vezeridis A. (2017). Contrast-enhanced ultrasound (CEUS) liver imaging reporting and data system (LI-RADS) 2017—A review of important differences compared to the CT/MRI system. Clin. Mol. Hepatol..

[7] Wilson S.R., Lyshchik A., Piscaglia F., Cosgrove D., Jang H.-J., Sirlin C., Dietrich C.F., Kim T.K., Willmann J.K., Kono Y. (2017). CEUS LI-RADS: Algorithm, implementation, and key differences from CT/MRI. Abdom. Radiol..

[8] Hu J., Bhayana D., Burak K.W., Wilson S.R. (2019). Resolution of indeterminate MRI with CEUS in patients at high risk for hepatocellular carcinoma. Abdom. Radiol..

[9] Kang H.-J., Kim J.H., Joo I., Han J.K. (2019). Additional value of contrast-enhanced ultrasound (CEUS) on arterial phase non-hyperenhancement observations (≥2 cm) of CT/MRI for high-risk patients: Focusing on the CT/MRI LI-RADS categories LR-3 and LR-4. Abdom. Radiol..

[10] Santillan C., Chernyak V., Sirlin C. (2018). LI-RADS categories: Concepts, definitions, and criteria. Abdom. Radiol..

[11] Heimbach J.K., Kulik L.M., Finn R.S., Sirlin C.B., Abecassis M.M., Roberts L.R., Zhu A.X., Murad M.H., Marrero J.A. (2018). AASLD guidelines for the treatment of hepatocellular carcinoma. Hepatology.

[12] Tang A., Bashir M.R., Corwin M.T., Cruite I., Dietrich C.F., Do R.K.G., Ehman E.C., Fowler K.J., Hussain H.K., Jha R.C. (2018). Evidence Supporting LI-RADS Major Features for CT- and MR Imaging-based Diagnosis of Hepatocellular Carcinoma: A Systematic Review. Radiology.

[13] Denecke T., Grieser C., Froling V., Steffen I.G., Rudolph B., Stelter L., Lehmkuhl L., Streitparth F., Langrehr J., Neuhaus P. (2009). Multislice computed tomography using a triple-phase contrast protocol for preoperative assessment of hepatic tumor load in patients with hepatocellular carcinoma before liver transplantation. Transpl. Int..

[14] Maruyama H., Takahashi M., Ishibashi H., Yoshikawa M., Yokosuka O. (2012). Contrast-enhanced ultrasound for characterisation of hepatic lesions appearing non-hypervascular on CT in chronic liver diseases. Br. J. Radiol..

[15] Takahashi M., Maruyama H., Shimada T., Kamezaki H., Sekimoto T., Kanai F., Yokosuka O. (2013). Characterization of hepatic lesions (≤30 mm) with liver-specific contrast agents: A comparison between ultrasound and magnetic resonance imaging. Eur. J. Radiol..

[16] Bolondi L., Gaiani S., Celli N., Golfieri R., Grigioni W.F., Leoni S., Venturi A.M., Piscaglia F. (2005). Characterization of small nodules in cirrhosis by assessment of vascularity: The problem of hypovascular hepatocellular carcinoma. Hepatology.

[17] Leoni S., Piscaglia F., Granito A., Borghi A., Galassi M., Marinelli S., Terzi E., Bolondi L. (2013). Characterization of primary and recurrent nodules in liver cirrhosis using contrast-enhanced ultrasound: Which vascular criteria should be adopted?. Ultraschall Med. Eur. J. Ultrasound.

[18] Jang H.J., Kim T.K., Khalili K., Yazdi L., Menezes R., Park S.H., Sherman M. (2013). Characterization of 1-to 2-cm liver nodules detected on hcc surveillance ultrasound according to the criteria of the American Association for the Study of Liver Disease: Is quadriphasic CT necessary?. Am. J. Roentgenol..

[19] Park M.J., Kim Y.K., Lee M.H., Lee J.H. (2013). Validation of diagnostic criteria using gadoxetic acid-enhanced and diffusion-weighted MR imaging for small hepatocellular carcinoma (≤2.0 cm) in patients with hepatitis-induced liver cirrhosis. Acta Radiol..

[20] Valls C., Cos M., Figueras J., Andia E., Ramos E., Sanchez A., Serrano T., Torras J. (2004). Pretransplantation diagnosis and staging of hepatocellular carcinoma in patients with cirrhosis: Value of dual-phase helical CT. Am. J. Roentgenol..

[21] Jang H.J., Kim T.K., Wilson S.R. (2009). Small nodules (1–2 cm) in liver cirrhosis: Characterization with contrast-enhanced ultrasound. Eur. J. Radiol..

[22] Giorgio A., Montesarchio L., Gatti P., Amendola F., Matteucci P., Santoro B., Merola M.G., Merola F., Coppola C., Giorgio V. (2016). Contrast-Enhanced Ultrasound: A Simple and Effective Tool in Defining a Rapid Diagnostic Work-up for Small Nodules Detected in Cirrhotic Patients during Surveillance. J. Gastrointest. Liver Dis..

[23] Elsayes K.M., Kielar A.Z., Agrons M.M., Szklaruk J., Tang A., Bashir M.R., Mitchell D.G., Do R.K., Fowler K.J., Chernyak V. (2017). Liver Imaging Reporting and Data System: An expert consensus statement. J. Hepatocell. Carcinoma.

[24] Sangiovanni A., Manini M.A., Iavarone M., Romeo R., Forzenigo L.V., Fraquelli M., Massironi S., Della Corte C., Ronchi G., Rumi M.G. (2010). The diagnostic and economic impact of contrast imaging techniques in the diagnosis of small hepatocellular carcinoma in cirrhosis. Gut.

[25] Kim T.K., Lee K.H., Jang H.J., Haider M.A., Jacks L.M., Menezes R.J., Park S.H., Yazdi L., Sherman M., Khalili K. (2011). Analysis of gadobenate dimeglumine-enhanced MR findings for characterizing small (1–2-cm) hepatic nodules in patients at high risk for hepatocellular carcinoma. Radiology.

[26] Rimola J., Forner A., Tremosini S., Reig M., Vilana R., Bianchi L., Rodriguez-Lope C., Sole M., Ayuso C., Bruix J. (2012). Non-invasive diagnosis of hepatocellular carcinoma ≤ 2 cm in cirrhosis. Diagnostic accuracy assessing fat, capsule and signal intensity at dynamic MRI. J. Hepatol..

[27] Wang J.Y., Feng S.Y., Yi A.J., Zhu D., Xu J.W., Li J., Cui X.W., Dietrich C.F. (2020). Comparison of Contrast-Enhanced Ultrasound versus Contrast-Enhanced Magnetic Resonance Imaging for the Diagnosis of Focal Liver Lesions Using the Liver Imaging Reporting and Data System. Ultrasound Med. Biol..

[28] Leoni S., Piscaglia F., Golfieri R., Camaggi V., Vidili G., Pini P., Bolondi L. (2010). The impact of vascular and nonvascular findings on the noninvasive diagnosis of small hepatocellular carcinoma based on the EASL and AASLD criteria. Am. J. Gastroenterol..

[29] Wilson S.R., Kim T.K., Jang H.J., Burns P.N. (2007). Enhancement patterns of focal liver masses: Discordance between contrast-enhanced sonography and contrast-enhanced CT and MRI. Am. J. Roentgenol..

[30] Burns P.N., Wilson S.R. (2007). Focal liver masses: Enhancement patterns on contrast-enhanced images—concordance of US scans with CT scans and MR images. Radiology.

[31] D’Onofrio M., Crosara S., De Robertis R., Canestrini S., Cantisani V., Morana G., Mucelli R.P. (2014). Malignant focal liver lesions at contrast-enhanced ultrasonography and magnetic resonance with hepatospecific contrast agent. Ultrasound.

[32] Han J., Liu Y., Han F., Li Q., Yan C., Zheng W., Wang J., Guo Z., Wang J., Li A. (2015). The Degree of Contrast Washout on Contrast-Enhanced Ultrasound in Distinguishing Intrahepatic Cholangiocarcinoma from Hepatocellular Carcinoma. Ultrasound Med. Biol..

[33] Chen L.D., Xu H.X., Xie X.Y., Xie X.H., Xu Z.F., Liu G.J., Wang Z., Lin M.X., Lu M.D. (2010). Intrahepatic cholangiocarcinoma and hepatocellular carcinoma: Differential diagnosis with contrast-enhanced ultrasound. Eur. Radiol..

[34] Dietrich C.F., Cui X.W., Boozari B., Hocke M., Ignee A. (2012). Contrast-enhanced ultrasound (CEUS) in the diagnostic algorithm of hepatocellular and cholangiocellular carcinoma, comments on the AASLD guidelines. Ultraschall Med..

[35] Li R., Yuan M.X., Ma K.S., Li X.W., Tang C.L., Zhang X.H., Guo D.Y., Yan X.C. (2014). Detailed analysis of temporal features on contrast enhanced ultrasound may help differentiate intrahepatic cholangiocarcinoma from hepatocellular carcinoma in cirrhosis. PLoS ONE.

[36] Wildner D., Bernatik T., Greis C., Seitz K., Neurath M.F., Strobel D. (2015). CEUS in hepatocellular carcinoma and intrahepatic cholangiocellular carcinoma in 320 patients—Early or late washout matters: A subanalysis of the DEGUM multicenter trial. Ultraschall Med..

[37] Terzi E., Iavarone M., Pompili M., Veronese L., Cabibbo G., Fraquelli M., Riccardi L., De Bonis L., Sangiovanni A., Leoni S. (2018). Contrast ultrasound LI-RADS LR-5 identifies hepatocellular carcinoma in cirrhosis in a multicenter restropective study of 1006 nodules. J. Hepatol..

[38] Kang Y., Lee J.M., Kim S.H., Han J.K., Choi B.I. (2012). Intrahepatic mass-forming cholangiocarcinoma: Enhancement patterns on gadoxetic acid-enhanced MR images. Radiology.

[39] Jeong H.T., Kim M.J., Chung Y.E., Choi J.Y., Park Y.N., Kim K.W. (2013). Gadoxetate disodium-enhanced MRI of mass-forming intrahepatic cholangiocarcinomas: Imaging-histologic correlation. Am. J. Roentgenol..

[40] Chong Y.S., Kim Y.K., Lee M.W., Kim S.H., Lee W.J., Rhim H.C., Lee S.J. (2012). Differentiating mass-forming intrahepatic cholangiocarcinoma from atypical hepatocellular carcinoma using gadoxetic acid-enhanced MRI. Clin. Radiol..

[41] Cerny M., Chernyak V., Olivie D., Billiard J.S., Murphy-Lavallee J., Kielar A.Z., Elsayes K.M., Bourque L., Hooker J.C., Sirlin C.B. (2018). LI-RADS Version 2018 Ancillary Features at MRI. Radiographics.

[42] Iavarone M., Piscaglia F., Vavassori S., Galassi M., Sangiovanni A., Venerandi L., Forzenigo L.V., Golfieri R., Bolondi L., Colombo M. (2013). Contrast enhanced CT-scan to diagnose intrahepatic cholangiocarcinoma in patients with cirrhosis. J. Hepatol..

[43] Kim S.J., Lee J.M., Han J.K., Kim K.H., Lee J.Y., Choi B.I. (2007). Peripheral mass-forming cholangiocarcinoma in cirrhotic liver. Am. J. Roentgenol..

[44] Kim S.A., Lee J.M., Lee K.B., Kim S.H., Yoon S.H., Han J.K., Choi B.I. (2011). Intrahepatic mass-forming cholangiocarcinomas: Enhancement patterns at multiphasic CT, with special emphasis on arterial enhancement pattern—correlation with clinicopathologic findings. Radiology.

[45] Chung Y.E., Kim M.J., Park Y.N., Choi J.Y., Pyo J.Y., Kim Y.C., Cho H.J., Kim K.A., Choi S.Y. (2009). Varying appearances of cholangiocarcinoma: Radiologic-pathologic correlation. Radiographics.

[46] Chernyak V., Kobi M., Flusberg M., Fruitman K.C., Sirlin C.B. (2017). Effect of threshold growth as a major feature on LI-RADS categorization. Abdom. Radiol..

[47] Elsayes K.M., Kielar A.Z., Chernyak V., Morshid A., Furlan A., Masch W.R., Marks R.M., Kamaya A., Do R.K.G., Kono Y. (2019). LI-RADS: A conceptual and historical review from its beginning to its recent integration into AASLD clinical practice guidance. J. Hepatocell. Carcinoma.

[48] Alhasan A., Cerny M., Olivie D., Billiard J.S., Bergeron C., Brown K., Bodson-Clermont P., Castel H., Turcotte S., Perreault P. (2019). LI-RADS for CT diagnosis of hepatocellular carcinoma: Performance of major and ancillary features. Abdom. Radiol..

[49] Choi D., Mitchell D.G., Verma S.K., Bergin D., Navarro V.J., Malliah A.B., McGowan C., Hann H.W., Herrine S.K. (2007). Hepatocellular carcinoma with indeterminate or false-negative findings at initial MR imaging: Effect on eligibility for curative treatment initial observations. Radiology.

[50] Taouli B., Goh J.S., Lu Y., Qayyum A., Yeh B.M., Merriman R.B., Coakley F.V. (2005). Growth rate of hepatocellular carcinoma: Evaluation with serial computed tomography or magnetic resonance imaging. J. Comput. Assist. Tomogr..

[51] Toyoda H., Kumada T., Honda T., Hayashi K., Katano Y., Nakano I., Hayakawa T., Fukuda Y. (2001). Analysis of hepatocellular carcinoma tumor growth detected in sustained responders to interferon in patients with chronic hepatitis C. J. Gastroenterol. Hepatol..

[52] Saito Y., Matsuzaki Y., Doi M., Sugitani T., Chiba T., Abei M., Shoda J., Tanaka N. (1998). Multiple regression analysis for assessing the growth of small hepatocellular carcinoma: The MIB-1 labeling index is the most effective parameter. J. Gastroenterol..

[53] Shingaki N., Tamai H., Mori Y., Moribata K., Enomoto S., Deguchi H., Ueda K., Inoue I., Maekita T., Iguchi M. (2013). Serological and histological indices of hepatocellular carcinoma and tumor volume doubling time. Mol. Clin. Oncol..

[54] Shimofusa R., Ueda T., Kishimoto T., Nakajima M., Yoshikawa M., Kondo F., Ito H. (2010). Magnetic resonance imaging of hepatocellular carcinoma: A pictorial review of novel insights into pathophysiological features revealed by magnetic resonance imaging. J. Hepatobiliary Pancreat. Sci..

[55] Ishigami K., Yoshimitsu K., Nishihara Y., Irie H., Asayama Y., Tajima T., Nishie A., Hirakawa M., Ushijima Y., Okamoto D. (2009). Hepatocellular carcinoma with a pseudocapsule on gadolinium-enhanced MR images: Correlation with histopathologic findings. Radiology.

[56] Cerny M., Bergeron C., Billiard J.S., Murphy-Lavallee J., Olivie D., Berube J., Fan B., Castel H., Turcotte S., Perreault P. (2018). LI-RADS for MR Imaging Diagnosis of Hepatocellular Carcinoma: Performance of Major and Ancillary Features. Radiology.

[57] Khan A.S., Hussain H.K., Johnson T.D., Weadock W.J., Pelletier S.J., Marrero J.A. (2010). Value of delayed hypointensity and delayed enhancing rim in magnetic resonance imaging diagnosis of small hepatocellular carcinoma in the cirrhotic liver. J. Magn. Reson. Imaging.

[58] Zhang Y.D., Zhu F.P., Xu X., Wang Q., Wu C.J., Liu X.S., Shi H.B. (2016). Liver Imaging Reporting and Data System: Substantial Discordance Between CT and MR for Imaging Classification of Hepatic Nodules. Acad. Radiol..

[59] Corwin M.T., Fananapazir G., Jin M., Lamba R., Bashir M.R. (2016). Differences in Liver Imaging and Reporting Data System Categorization Between MRI and CT. Am. J. Roentgenol..

[60] Kadoya M., Matsui O., Takashima T., Nonomura A. (1992). Hepatocellular carcinoma: Correlation of MR imaging and histopathologic findings. Radiology.

[61] Khatri G., Merrick L., Miller F.H. (2010). MR imaging of hepatocellular carcinoma. Magn. Reson. Imaging Clin. N. Am..

[62] Mitchell D.G., Rubin R., Siegelman E.S., Burk D.L., Rifkin M.D. (1991). Hepatocellular carcinoma within siderotic regenerative nodules: Appearance as a nodule within a nodule on MR images. Radiology.

[63] Cruite I., Santillan C., Mamidipalli A., Shah A., Tang A., Sirlin C.B. (2016). Liver Imaging Reporting and Data System: Review of Ancillary Imaging Features. Semin. Roentgenol..

[64] Choi S.H., Byun J.H., Kim S.Y., Lee S.J., Won H.J., Shin Y.M., Kim P.N. (2016). Liver Imaging Reporting and Data System v2014 With Gadoxetate Disodium-Enhanced Magnetic Resonance Imaging: Validation of LI-RADS Category 4 and 5 Criteria. Investig. Radiol..

[65] Darnell A., Forner A., Rimola J., Reig M., Garcia-Criado A., Ayuso C., Bruix J. (2015). Liver Imaging Reporting and Data System with MR Imaging: Evaluation in Nodules 20 mm or Smaller Detected in Cirrhosis at Screening US. Radiology.

[66] American College of Radiology Ultrasound LI-RADS v2017. https://www.acr.org/Clinical-Resources/Reporting-and-Data-Systems/LI-RADS/Ultrasound-LI-RADS-v2017.

[67] Moudgil S., Kalra N., Prabhakar N., Dhiman R.K., Behera A., Chawla Y.K., Khandelwal N. (2017). Comparison of Contrast Enhanced Ultrasound with Contrast Enhanced Computed Tomography for the Diagnosis of Hepatocellular Carcinoma. J. Clin. Exp. Hepatol..

[68] Xu J.F., Liu H.Y., Shi Y., Wei Z.H., Wu Y. (2011). Evaluation of hepatocellular carcinoma by contrast-enhanced sonography: Correlation with pathologic differentiation. J. Ultrasound Med..

[69] Gambarin-Gelwan M., Wolf D.C., Shapiro R., Schwartz M.E., Min A.D. (2000). Sensitivity of commonly available screening tests in detecting hepatocellular carcinoma in cirrhotic patients undergoing liver transplantation. Am. J. Gastroenterol..

[70] Claudon M., Dietrich C.F., Choi B.I., Cosgrove D.O., Kudo M., Nolsoe C.P., Piscaglia F., Wilson S.R., Barr R.G., Chammas M.C. (2013). Guidelines and good clinical practice recommendations for contrast enhanced ultrasound (CEUS) in the liver--update 2012: A WFUMB-EFSUMB initiative in cooperation with representatives of AFSUMB, AIUM, ASUM, FLAUS and ICUS. Ultraschall Med..

[71] Tzartzeva K., Obi J., Rich N.E., Parikh N.D., Marrero J.A., Yopp A., Waljee A.K., Singal A.G. (2018). Surveillance Imaging and Alpha Fetoprotein for Early Detection of Hepatocellular Carcinoma in Patients with Cirrhosis: A Meta-analysis. Gastroenterology.

[72] Singal A., Volk M.L., Waljee A., Salgia R., Higgins P., Rogers M.A., Marrero J.A. (2009). Meta-analysis: Surveillance with ultrasound for early-stage hepatocellular carcinoma in patients with cirrhosis. Aliment. Pharm. Ther..

[73] Chou R., Cuevas C., Fu R., Devine B., Wasson N., Ginsburg A., Zakher B., Pappas M., Graham E., Sullivan S.D. (2015). Imaging Techniques for the Diagnosis of Hepatocellular Carcinoma: A Systematic Review and Meta-analysis. Ann. Intern. Med..

